# Q&A: The brain under a mesoscope: the forest and the trees

**DOI:** 10.1186/s12915-017-0426-y

**Published:** 2017-09-14

**Authors:** Nicholas James Sofroniew

**Affiliations:** Janelia Research Campus, Ashburn, VA 20147 USA

## Abstract

Neurons relevant to a particular behavior are often widely dispersed across the brain. To record activity in groups of individual neurons that might be distributed across large distances, neuroscientists and optical engineers have been developing a new type of microscope called a mesoscope. Mesoscopes have high spatial resolution and a large field of view. This Q&A will discuss this exciting new technology, highlighting a particular instrument, the two-photon random access mesoscope (2pRAM).

## What does a brain look like under a mesoscope?

A mouse brain contains 100 million neurons. Individual neurons have cell bodies with diameters on the order of 10 μm. Neurons relevant to any given behavior are distributed over millimeters of tissue and form complex networks via billions of synapses, over both short and long (>1 mm) distances. One major mystery is how the coordinated activity in large populations of neurons drives behavior. Another mystery is how the neuronal activity patterns emerge as a result of structured synaptic connectivity. Recording the activity in large populations of neurons during behavior promises to shed light on both mysteries.

A major new enabling technology is the development of genetically encoded indicators of calcium (GECI), based on fluorescent proteins fused to a calcium binding domain [1]. When a neuron is quiescent its intracellular resting calcium is low. When the neuron fires an action potential, calcium levels rise by a factor of ten, which can be recorded as a change in fluorescence of the GECI. Microscopy together with calcium imaging already allows us to record movies of neuronal activity, but with important limitations on the size of the imaged structures.

Higher magnification and better spatial resolution typically imply a smaller field of view. Commercial microscopes with cellular resolution typically have small (<1 mm) fields of view and cannot simultaneously image activity distributed across multiple brain areas. Conversely, macroscopic imaging methods can have large fields of view (>10 mm) to capture many different brain areas simultaneously, but cannot resolve single cells. These macroscopic methods indiscriminately mix together signals from neurons with different properties. However, the resolution limit is fundamentally independent of magnification and field of view. The inverse relationship between field of view and resolution instead is caused by constraints imposed by design principles for general-purpose objectives.

**Fig. 1.**
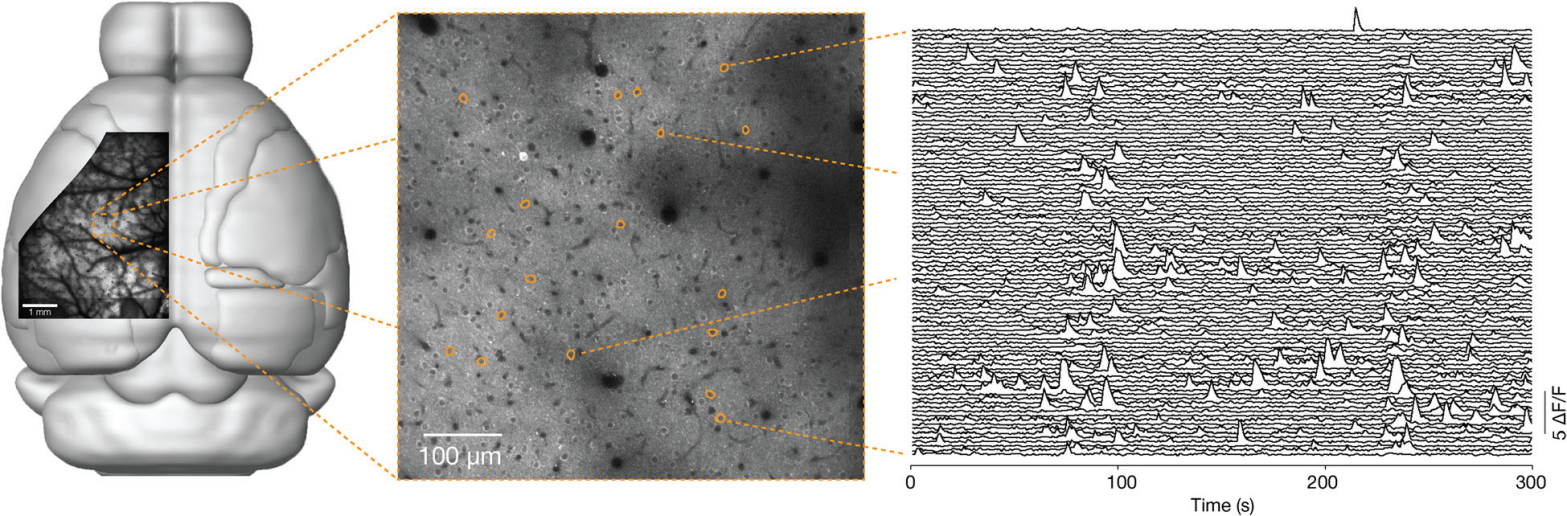
*Left*: A field of view from the 2pRAM overlaid on a mouse brain. The 2pRAM can capture a large fraction of the accessible surface of the mouse cortex. *Center*: A zoom into a small portion of the field of view. Individual neurons are visible as *donuts. Right*: Neural activity of individual neurons

The term mesoscope refers to a new type of optical instrument with large fields of view, but with subcellular resolution. The two-photon random access mesoscope (2pRAM) has a field of view of 5 mm, corresponding to many brain areas in the mouse, and a spatial resolution of < 5 μm, to isolate signals from individual neurons (Fig. 1). These instruments allow us to see both the proverbial forest and the trees.

## Why do we need mesoscopes in neuroscience?

Much of our understanding in neuroscience is of the properties of individual neurons and their subcellular compartments or the average properties of brain areas. Much less is known about how neurons interact in neural circuits across multiple brain areas to give rise to brain function. Conventional methods miss key information about the function of the underlying neural circuits. In the case of monitoring a small number of individual neurons in one brain area, measuring correlations and interactions between far away neurons is impossible. In the case of monitoring population average activity, determining the functional properties of individual neurons is impossible. Mesoscope technology allows for the measurement of both the functional properties of and the correlations between individual widely separated neurons. These data will be crucial for deciphering how neural circuits function.

In addition, a worthy goal of a new technology is to reduce the number of animals used for a study. By having larger fields of view compared with conventional microscopes a mesoscope enables more data to be collected per animal. More data enables more meaningful comparisons within the same animal, thereby reducing the numbers of animals needed per study.

## What are examples of mesoscopes?

A mesoscope is a type of microscope with both a fine spatial resolution (<5 μm) for resolving single cells and a large field of view (>5 mm) to detect neurons across a large area. Achieving these specifications requires specialized optics, including microscope objectives, with a high numerical aperture and low magnification. Such custom “mesolenses” have been developed for confocal microscopy [2], and two-photon microscopy, for example, the Trepan2p system [3], the ultra-large field-of-view system [4], and the 2pRAM system [5]. Two-photon microscopy allows for imaging deep into scattering tissue [6]. The Ultra-large FOV, Trepan2p, and 2pRAM systems rely on two-photon excitation and are therefore well-suited for imaging neural activity. The Ultra-large FOV is built with commercial components and achieves 1 μm lateral resolution across 10 mm using a 0.28 numerical aperture objective. The Trepan2p has a custom objective with numerical aperture of 0.43 and field of view of 3.5 mm. The 2pRAM has a custom objective with numerical aperture of 0.6 and a field of view of 5 mm. The 2pRAM system allows for random access imaging across this large field of view.

## What animal species are mesoscopes good for?

Most calcium imaging experiments using mesoscopes are currently performed in mice. However, experiments in rats are also routine. Furthermore, several projects are ongoing to adapt these methods to studies of the primate brain.

## How are images created over such large fields of view?

The 2pRAM uses two-photon excitation to create image contrast. This system builds up images in time by scanning a single point of excitation, at a laser beam’s focus, over the sample and collecting the emitted light using a point detector. The detector is a photomultiplier tube that allows detection of very weak signals. The speed and flexibility of imaging is determined by the scan system that is used to position the focus of the beam. Rather than a traditional scan system containing only a single xy-galvo pair of scan mirrors, the 2pRAM contains a serial scan system where multiple scan mirrors are used to allow for different imaging acquisition configurations. The beam first hits a fast resonant scanner (12 kHz). This mirror oscillates at a fixed frequency, and for a given imaging experiment, a fixed amplitude. It is used to create a very fast line of excitation that can then be positioned arbitrarily within the field of view using a slower xy-galvo pair. This arrangement allows for fast high magnification imaging to be performed over small areas that are far apart in a random access manner, or low magnification imaging over the entire field by tiling scans.

## Is there a tradeoff in the number of neurons that can be imaged and the signal that can be recovered?

Yes, parameters like overall frame rate, pixel size, pixel dwell time, and area imaged can be traded off against each other depending on the application. Some typical scan configurations are provided in Table 1.

**Table 1:** Frame rates for scanning different size fields of view at different spatial resolutions using the 2pRAM

Scan area (mm^2^)	Pixel size (μm^2^)	Number of regions of interest	Frame rate (Hz)
4.8 × 4.8	10 × 10	1	4.6
4.8 × 4.8	1 × 1	1	0.6
2.4 × 2.4	2 × 2	1	4.3
2.4 × 2.4	1 × 1	1	2.3
1.2 × 1.2	1 × 1	1	8.9
1.2 × 1.2	1 × 1	2	4.6
0.6 × 0.6	1 × 1	1	40.0
0.6 × 0.6	1 × 1	4	8.1

## Brains aren’t flat. How does the 2pRAM image different depths?

Neural activity related to specific functions occurs at different depths in different areas. To track neural activity at different depths many microscopes move either the objective or the sample in the vertical (z) dimension. This strategy is too slow for large samples or large objectives, like those used in a mesoscope. For rapid scanning the 2pRAM contains a remote focus unit [7]. This unit uses a small mirror to control the convergence and divergence of the beam into the main objective. By repositioning this mirror the position of the beam’s focus in z can be rapidly controlled. The addition of a custom remote focus objective allows for the z-scanning to be done in an aberration-free manner and results in superior performance compared to other remote focusing elements. In the 2pRAM the remote focus range is 1 mm, and a rapid and light-weight voice coil-driven linear stage allows for steps of 500 μm in 6 ms. Different locations in the brain can be viewed in a random access manner.

## What are the consequences of having a remote focus unit on the 2pRAM?

The remote focus unit allows for rapid positioning of the focus in the z-dimension. This feature also allows the 2pRAM objective to have a curved field of view (125 μm z-offset at the edges of the 5 mm) as it can be compensated for during scanning by moving the remote focus mirror. Allowing for a curved field greatly simplifies the design and number of elements in the optical system. The addition of the remote focusing means that the distances between all the optical elements have to remain fixed, as the shape of the optical wavefront would vary at different positions of the remote focus mirror. As a result, the system was designed to share aberration corrections between all the objectives and pupil relays. A consequence of this design is that all the optical elements were mounted in fixed positions on a single vertical breadboard.

## How deep can be imaged?

Although the 2pRAM is corrected over a depth range of 1 mm, the actual imaging depth is determined by scattering of excitation and the expression pattern of the indicator. Under standard conditions the depth limit is around 600 μm deep into the cortex, similar to standard two-photon microscopy. This range allows for imaging down to superficial layer 5 of the mouse cortex.

## How many neurons can be simultaneously imaged?

We have detected over 200,000 individual nuclei up to 600 μm deep in the 5 mm diameter field of view. A fraction of neurons are obstructed by vascular shading. Of the 200,000 neurons, only a subset can be imaged simultaneously. The exact number depends on the desired spatial and temporal resolution. Imaging across a 4 × 4 mm^2^ area at just over 2 μm pixel resolution and at 2 Hz contained over 3000 active neurons, and many silent neurons. As computational methods improve for detecting neurons in noisy images, this number will increase.

## Is there a possibility for multiplexing to increase acquisition rates?

Temporal multiplexing with two beams could double the acquisition rate as is done in the Trepan2 system. Spatial multiplexing with an array of beamlets could also increase the acquisition rate. However, the total signal collected is limited by the laser power that can be introduced into the brain without heating. For fixed total power, multiplexing reduces the efficiency of signal generation in proportion to the multiplexing. If the goal is to maximize the number of recorded neurons, then a single beam scanned rapidly is preferred.

## What are some limitations of the mesoscope?

The 2pRAM is very large and mounted on a vertical breadboard that is a meter long, making it a challenge to move. This breadboard is moved by a gantry system that in turn takes up a lot of space (4’ × 5’ × 3’). If multi-area recordings are not required, then a conventional two-photon microscope should be used instead. The large size and form factor of the objective makes simultaneous electrophysiological measurements difficult, as access for pipettes is blocked.

## What are some possible extensions of the mesoscope?

The mesoscope could be used in conjunction with a GRIN lens or a prism for deeper tissue imaging or imaging areas that are otherwise hard to access.

The mesoscope also has space for a second beam path to be coupled into the main objective. This beam path can be used for optogenetic manipulations to look at the causal effect of neural activity on behavior [8]. A possible experiment would be to inactivate one brain area while recording the effect on individual neurons in a downstream area. Combined manipulation and recording experiments could help elucidate how activity moves through neural circuits.
